# Positive outcomes of implementing applied theatrical improvisation in communication trainings/ workshops for healthcare students in two European countries: a comparative study

**DOI:** 10.1192/j.eurpsy.2024.390

**Published:** 2024-08-27

**Authors:** J. D. Fekete, M. Jouin, K. Eklicsné Lepenye, Z. Pótó, M. Hainselin

**Affiliations:** ^1^Department of Languages for Biomedical Purposes and Communication, University of Pécs Medical School, Pécs, Hungary; ^2^Bureau E319bis - Campus Sud Psychologie Santé, Université de Picardie Jules Verne, Amiens, France; ^3^Institute of Psysiotherapy and sportsciences, University of Pécs Medical School Faculty of Health Sciences, Pécs, Hungary

## Abstract

**Introduction:**

Effective communication has been shown to improve patients’ health outcomes. This study utilizes medical improvisation techniques to teach communication skills to different groups of health students (nurses, midwives, medical doctors, speech therapists).

**Objectives:**

Our objective was to design and compare an interprofessional workshop that incorporates applied improvisation to train different groups of healthcare students in communication skills, resilience, dealing with failure and empathy. Medical improvisation is an innovative concept to prepare healthcare students to be more effective communicators.

**Methods:**

Required medical improv workshops (using applied improvisational theater techniques) were held for first to third-year students in France and in Hungary. Workshop evaluations were obtained before and following the last session and at 3 months post-workshop for one cohort. The courses incorporated role plays, listening, storytelling and verbal/ nonverbal exercises to help students communicate with empathy and clarity. The two countries used the same questionnaires for assessment (Interpersonal Communication Questionnaire and Intolerance Uncertainty Scale)

**Results:**

24 medical students participated in the Hungarian improvisation workshops, and 26 speech therapists students in the French improvisation workshops. In the finished Hungarian research over 90% of students rated the workshops as above average or excellent. Students reported a gain in insights regarding their role as a clinician (≥ 90%), an improvement in their ability to demonstrate effective communication (80–87%), and a positive impact on teamwork (91–93%). At 3 months post-workshop, students reported they had used at least 1 improvisation skill on their clinical wards. Both countries can claim promising results so far in their separate studies, our results comparing the French and Hungarian data using synchronized scales and questionnaires is currently in progress, and will be processed by the end of this year.

**Conclusions:**

This study demonstrates that medical improvisation exercises can be scaled to different fields of healthcare students in various years of their studies and that using improv in healthcare education is universal in its short and long-term effects. Further, we found that students felt that it improved their communication. This study also provides new insights regarding specific improvisation exercises that are most useful for the clinical environment.

Keywords: Improvisation. Medical improvisation. Applied Improvisation. Medical education. Communication. Uncertainty tolerance. Soft skills training

**Disclosure of Interest:**

None Declared

